# Strokeformer: A novel deep learning paradigm training transformer-based architecture for stroke prognosis prediction

**DOI:** 10.1371/journal.pone.0330530

**Published:** 2025-08-26

**Authors:** Maocheng Cao, Haochang Jin, Yuxi Wang, Li Wang, Junkai Ji

**Affiliations:** 1 Shenzhen Fuyong People’s Hospital, Shenzhen, China; 2 National Engineering Laboratory for Big Data System Computing Technology, Shenzhen University, Shenzhen, China; 3 Department of Computer Science, City University of Hong Kong, Hong Kong, China; 4 Department of Neurology, The Affiliated Taizhou People’s Hospital of Nanjing Medical University, Taizhou, Jiangsu, China; Majmaah University, SAUDI ARABIA

## Abstract

Stroke, a common neurological disorder, is considered one of the leading causes of death and disability worldwide. Stroke prognosis issues involve using clinical characteristics collected from patients presented in tabular form to determine whether they are suitable for thrombolytic therapy. Transformer-based deep learning methods have achieved state-of-the-art performance in various classification tasks, but flaws still exist in dealing with tabular data. These models and algorithms largely tend to overfit and exhibit performance degeneration on small-scale, class-imbalanced datasets. Medical datasets are typically small and imbalanced due to the scarcity of labelled medical data samples. Therefore, this study proposes a novel stroke prognosis prediction model called Strokeformer to address these issues. Specifically, novel intra- and interfeature interaction modules are designed to capture internal and mutual information among individual features for more effective latent representations. In addition, we explore the possibility of performing the training process by pretraining on large-scale, class-balanced datasets and then fine-tuning on small-scale, class-imbalanced downstream datasets. This pretraining and fine-tuning paradigm is dramatically feasible for preventing overfitting. To verify the effectiveness of the proposed model and training method, experiments are conducted on 20 public datasets from OpenML and two private stroke prognosis datasets provided by Shenzhen Fuyong People’s Hospital and The Affiliated Taizhou People’s Hospital of Nanjing Medical University, China, respectively. The results show that Strokeformer performance significantly outperforms that of other comparison models on the introduced datasets. The principal limitation of the model lies in its lack of interpretability from the clinicians’ perspective. Nevertheless, given that the interpretability of deep learning remains an open challenge, the promising empirical results achieved by Strokeformer on real-world stroke prognosis datasets highlight its potential to assist in clinical decision-making.

## Introduction

Stroke is one of the leading causes of disability and death worldwide [1]. The prediction is that 12 million stroke deaths and more than 200 million disability-adjusted life-years could be lost from stroke each year by 2030 [2]. Stroke can be categorized into two major types depending on the cause: ischaemic stroke caused by vascular blockage and haemorrhagic stroke resulting from the rupture of a blood vessel. Among these, ischaemic stroke is the most common, accounting for approximately 71% of all stroke cases [3]. Ischaemic stroke disrupts the blood supply to a specific area of the brain, which causes damage to and death of neurons, leading to the loss of speech, motor skills, memory, and cognitive functions [4]. Patients may experience limb paralysis, difficulty walking, and problems swallowing. Additionally, cognitive and emotional aspects can also be affected, manifesting as memory decline, a lack of concentration, depression, and anxiety. In severe cases, it can be lethal [5].

The standard treatment for ischaemic stroke is thrombolytic therapy, which involves the use of thrombolytic agents to dissolve blood clots obstructing blood vessels and restoring cerebral blood flow. This therapy must be administered within a critical time window, typically within a few hours after the onset of stroke, to achieve optimal results [6]. In addition, not all patients are suitable candidates for this treatment, even within the time window [7]. Therefore, a comprehensive patient evaluation is needed before treatment to minimize risks. In the early stages of stroke prognosis, scoring methods are commonly used for clinical decision-making, which predict the patient outcome based on their clinical characteristics at the time of admission [8,9]. These methods are typically rule-based and developed from data specific to certain populations, resulting in poor generalization capabilities when applied to different populations. The application of artificial intelligence (AI) technologies has provided new approaches for stroke prognosis. By leveraging vast amounts of patient data, specifically developed AI models and algorithms have been demonstrated to help identify the intricate patterns and factors influencing stroke recovery, thereby increasing the accuracy of prognostic predictions.

Since stroke prognosis is essentially a data classification problem, many machine learning methods have been applied in the field of stroke prognosis prediction. Various machine learning techniques, including logistic regression (LR) [10], support vector machines (SVMs) [11], and random forests (RFs) [12], are employed to predict functional outcomes in patients with ischaemic stroke [13]. Their findings demonstrated that these machine-learning approaches significantly outperformed rule-based scoring systems. In a previous study [14], three distinct machine learning models, artificial neural networks (ANNs), LRs, and RFs, were utilized to predict long-term outcomes in patients with acute ischaemic stroke. This research revealed that the ANN outperformed both the RF and the LR. SVMs, RFs, and ANNs have been used by [15] to analyse the data collected by the Taiwan Stroke Registry since 2006. After 206 clinical variables were selected, 17 key features from the ischaemic stroke dataset and 22 features from the haemorrhagic stroke dataset were identified. LR, RF, and XGBoost [16] were employed to analyse data from 4,237 acute ischaemic stroke patients in [17]. The clinical data and CT brain images of 116 acute ischaemic stroke patients were analysed by [18] to train an SVM aimed at predicting symptomatic intracranial haemorrhage. The results demonstrated that the SVM significantly outperformed traditional scoring systems such as SEDAN [19] and HAT [20]. However, machine learning methods often rely on feature engineering [21], which is not only time-consuming but also requires substantial expertise. Additionally, machine learning methods have limitations in capturing complex data relationships [22,23].

In contrast, deep learning models, with their multilayer structures and nonlinear activation functions, can learn more complex data patterns and relationships [24–26]. Therefore, researchers have attempted to apply deep learning methods to the domain of stroke prognosis prediction. Deep convolutional neural networks are employed to predict the final lesion volume in stroke patients [27]. This model was capable of automatically identifying and integrating acute-phase imaging features, thereby enhancing predictive accuracy. Furthermore, the benefits of combining clinical data with neuroimaging features for predicting the three-month prognosis of acute ischaemic stroke patients have been demonstrated [28]. With the swift development of deep learning in recent years, more effective models have been proposed for tabular data classification problems. The transformer [29] is an innovative deep learning architecture that has significantly advanced in various fields, such as natural language processing [30] and image recognition [31]. This model has also been applied to tabular data classification problems [32]. Although the introduction of machine learning and deep learning methods has greatly improved the performance of stroke prognosis prediction models, conventional learning systems still suffer from problems such as limited data size and weak generalization capabilities because of the class-imbalanced training set.

Therefore, in this study, a novel transformer-based deep learning model named Strokeformer is proposed to address the problems described above. Strokeformer includes a feature embedding layer, a feature interaction layer, and an output layer. The feature embedding layer performs encoding of the input data. The feature interaction layer consists of an intrafeature interaction module and an interfeature interaction module, where the intrafeature interaction module applies a linear layer to individual feature vectors to learn information within each feature, and the interfeature interaction module contains several transformer encoder layers to capture the information between feature embeddings. The output layer projects the features learned from the former structure through a linear layer to obtain the final prediction result. Furthermore, this study applies a pretraining and fine-tuning learning paradigm in response to the problems of overfitting and performance deterioration caused by data imbalance. A large amount of unlabelled tabular data is first used for self-supervised learning in the pretraining phase, and then the small-scale target dataset with labelled samples is utilized for fine-tuning. This pretraining and fine-tuning method can effectively enhance the generalization ability of the model and alleviate overfitting on small-scale, class-imbalanced datasets. Experiments are performed on 19 benchmark tabular datasets and two real-world stroke prognosis datasets. The results show that, compared with other competitive methods, the proposed model and learning algorithm significantly improve the prediction results on the stroke prognosis dataset. In summary, the main contributions of this study can be summarized as follows:

- A novel Strokeformer model is introduced for stroke prognosis prediction, which contains customized intrafeature and interfeature interaction modules to capture internal and mutual information among individual features.
- A sophisticated learning paradigm is proposed to train Strokeformer to overcome overfitting and class imbalance issues, including a pretraining process on a large-scale, balanced dataset and a fine-tuning process on the downstream dataset.
- Extensive experiments have demonstrated that the proposed Strokeformer outperforms other state-of-the-art machine-learning and deep-learning methods on both benchmarks and two real-world stroke prognosis tasks.

The rest of the paper is organized as follows. The corresponding research on transformer-based models for medical data analysis and the latest machine-learning and deep-learning methods for tabular data classification tasks is summarized in the Related works section. The links of the code and the data are provided in the Materials and ethics statement section. The structural design and dataflow of the proposed Strokeformer model are described in the Model architecture section. The training algorithm, consisting of a self-supervised pretraining phase and a supervised fine-tuning phase, is illustrated in the Learning paradigm section. The experimental results of the proposed model and algorithm compared with those of conventional methods on all the introduced datasets are presented in the Experimental section. The Discussion section provides a discussion of some implementation details of the proposed model and algorithm, including an ablation study of the novel intrainteraction module and a sensitivity analysis of some variants in the model. Finally, the conclusions are given in the Conclusions section.

## Related works

On the one hand, the application of the transformer model in medical data analysis is explored. Transformer models have been widely applied in this field and have been successfully employed in various tasks, including image synthesis and reconstruction, registration, segmentation, detection, and diagnosis [33]. The Vision Transformer (ViT) [31] was further advanced by introducing a novel visual attention mechanism and achieved optimal performance on the COVID-19 diagnostic task without requiring pretraining on ImageNet [34]. By investigating the impact of image patch size when using ViT for tasks such as lesion lung classification and COVID-19 diagnosis, [35] revealed that increasing the patch size led to a decline in model performance, highlighting the trade-off between local and global information. A global-local transformer model designed for rapid brain age estimation using magnetic resonance imaging was proposed in [36]. The model consists of a global pathway that extracts contextual information from the entire input image and a local pathway that captures fine-grained details from image patches. Compared with other models, this approach significantly enhances the accuracy of brain age prediction. A transformer-based representation learning model designed to assist in clinical diagnosis was proposed in [37]. The model is capable of handling multimodal inputs, including patient X-rays, unstructured chief complaints, and structured clinical history data. Bidirectional intramodal and cross-modal attention layers are employed to learn comprehensive representations. This model outperforms image-only and nonintegrated multimodal diagnostic models in tasks such as identifying lung disease and predicting adverse clinical outcomes in COVID-19 patients.

On the other hand, state-of-the-art existing machine learning and deep learning-based tabular data classification solutions are also investigated in this study. Several classic methods are employed as competitors of the proposed model and algorithm in our experiments. XGBoost is an efficient machine-learning algorithm based on gradient-boosting decision trees. It uses a precise approximation method for finding split points and regularization techniques, which effectively avoids overfitting while enhancing the predictive accuracy and inference speed. CatBoost is also a gradient-boosting decision tree-based algorithm that automatically handles categorical features without requiring complex preprocessing. The algorithm introduces symmetric trees as base models, using identical splitting conditions at each layer, which accelerates the training speed and enhances the generalization ability [38]. Adaptive relation modeling network (ARMNet) transforms input features into the exponential space and dynamically sets cross-order and cross-weights for each feature, selectively modelling relationships between features effectively and capable of handling arbitrary-order cross features [39]. Neural network architecture for tabular data (TabNet) employs a multistep decision-making mechanism, selecting features for inference at each step through a sequential attention mechanism, thereby achieving interpretability of the model while enhancing learning efficiency [40]. The transformer architecture to tabular data (TabTransformer) model leverages the transformer structure based on self-attention, converting categorical feature embeddings into context-rich embeddings and thereby improving the predictive accuracy in supervised and semisupervised learning scenarios. The TabTranSELU is a simple yet effective adaptation of the transformer architecture for tabular data. The features alongside their respective names are encoded and fed into an enhanced transformer structure with scaled exponential linear unit activation [41]. Saint combines self-attention and intersample attention to capture complex interactions across features and data points, and both transformer blocks are helpful in advancing scalability and reducing computational overhead [42]. MambaTab is a recent innovative approach for tabular data that is based on an emerging structured state-space model variant named Mamba. It is indicated to have strong capabilities for efficiently extracting effective representations from data with long-range dependencies [43]. The Feature-Tokenizer Transformer (FT-Transformer) model combines feature transformation with the transformer architecture, differentially processing categorical and numerical features, achieving superior performance [44].

## Materials and ethics statement

Codes of the Strokeformer model and the pretraining and fine-tuning learning paradigm for it are available via https://github.com/jhc050998/Strokeformer. The two private stroke prognosis datasets used in this paper are available via https://ieee-dataport.org/documents/stroke-prognosis-dataset-taizhou-and-fuyong. The datasets were accessed on October 27, 2024. All the medical records were fully anonymized before we accessed them. The authors do not have access to information that could identify individual participants during or after data collection. This research has passed the formal ethical review process. We received official ethical approval letters such as Ethical Review Approval Letter with No. KY-2024-35 from Shenzhen Fuyong People’s Hospital and Ethical Review Committee Approval Letter with No. KY 2024-018-01 from the Clinical Research Ethics Committee of Taizhou People’s Hospital.

## Model architecture

**Fig 1:**
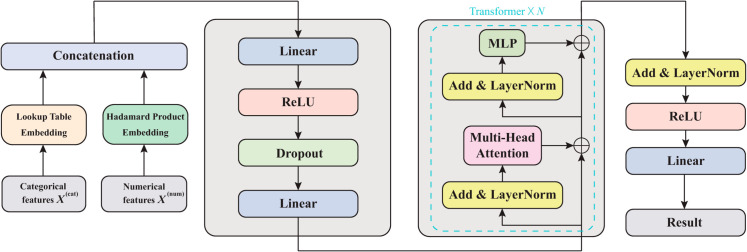
Description of the model architecture of Strokeformer. The model contains a feature embedding layer, a feature interaction layer consisting of an intrafeature interaction module, an interfeature interaction module, and an output layer that outputs the prediction result.

As shown in Fig 1, the Strokeformer architecture comprises three main parts: the feature embedding layer, the feature interaction layer, and the output layer. The feature embedding layer adopts two different encoding methods for categorical and numerical features. The feature interaction layer consists of an intrafeature interaction module followed by an interfeature interaction module. A stack of transformer layers is built in the interfeature interaction module, each consisting of a multihead self-attention layer followed by a positionwise feedforward layer. The output layer contains a linear layer to provide the final prediction result.

### Dataflow overview

Let (*X*,*y*) denote a feature–target pair, where $X=[X^{\left(num\right)},X^{\left(cat\right)}]$. $X^{\left(num\right)}\in {\mathbb{R}}^{d\times n}$ denotes all the *n* numerical features, and ${\text{X}}^{\left(\text{cat}\right)}$ denotes all of the *m* categorical features. For the embedding of numerical features, the Hadamard product is used to multiply ${\text{X}}^{\left(\text{num}\right)}$ with a parameter matrix $W\in {\mathbb{R}}^{d\times n}$, and a learnable bias matrix $B^{\left(num\right)}\in {\mathbb{R}}^{d\times n}$ is added to improve the capacity of the model. ${\text{T}}^{\left(\text{num}\right)}$ represents the matrix of embeddings for all the numerical features. Let $X^{\left(cat\right)}=[X_{1}^{\left(cat\right)},X_{2}^{\left(cat\right)},\cdots ,X_{m}^{\left(cat\right)}]$ with each $X_{i}^{\left(cat\right)}$ being a categorical feature, for i∈{1,⋯,m}. Each of the categorical features is embedded into a parametric embedding of dimension *d* using a lookup table approach, which is explained below in detail. Let $T_{i}^{\left(cat\right)}=E_{i}(X_{i}^{\left(cat\right)})\;+\;B_{i}^{\left(cat\right)}\in {\mathbb{R}}^{d}$ for i∈{1,⋯,m} be the embedding of the $X_{i}^{\left(cat\right)}$ feature and $T^{\left(cat\right)}=[T_{1}^{\left(cat\right)},T_{2}^{\left(cat\right)},\cdots ,T_{m}^{\left(cat\right)}]$ be the matrix of embeddings for all the categorical features.

Then, the numerical embeddings ${\text{T}}^{\left(\text{num}\right)}$ are concatenated along with the categorical embeddings ${\text{T}}^{\left(\text{cat}\right)}$ to form an d×(n+m) matrix *T*. The matrix of all the parametric embeddings is input into the intrafeature interaction module, which contains a multilayer perceptron (MLP) structure, to learn the information within each feature. Next, the output of the intrafeature interaction module is input into the interfeature interaction module consisting of *N* transformer layers followed by a feedforward layer. Each parametric embedding is transformed into contextual embedding when output from the interfeature interaction module through successive aggregation of context from other embeddings. We denote the intrafeature interaction module as a function $f_{\phi }^{\left(intra\right)}$ and the interfeature interaction module as $f_{\psi }^{\left(inter\right)}$. The functions $f_{\phi }^{\left(intra\right)}$ and $f_{\psi }^{\left(inter\right)}$ operate on parametric embeddings $T\in {\mathbb{R}}^{d\times (n+m)}$ in order and return corresponding contextual embeddings. These embeddings are input into an MLP, denoted by $g_{\theta }$, to predict the target *y*.

Let *H* be the cross-entropy for classification tasks. The following loss function *L*(*X*,*y*) is minimized to learn all the Strokeformer parameters via end-to-end learning by the first-order gradient methods. The Strokeformer parameters include *W*, ${\text{B}}^{\left(\text{num}\right)}$, and ${\text{B}}^{\left(\text{cat}\right)}$ for the feature embedding layer; *ϕ* and *ψ* for the feature interaction layer; and *θ* for the top MLP structure in the output layer. The overall loss function can be described as follows:

$$L(X,y)=H(g_{\theta }(f_{\psi }^{\left(inter\right)}(f_{\phi }^{\left(intra\right)}(concat(T^{\left(num\right)},T^{\left(cat\right)})))),y).$$ (1)

Below, more implementation details of each layer and module are explained.

### Feature embedding layer

Considering that tabular data typically include two different kinds of features, numerical features and categorical features, Strokeformer adopts different embedding methods for these two types of features. On the one hand, for numerical features, embedding via the Hadamard product is described as follows:

$$T^{\left(num\right)}=W⊙X^{\left(num\right)}+B^{\left(num\right)},$$ (2)

where ${\text{X}}^{\left(\text{num}\right)}$ is the matrix of raw numerical features in the input data and where ${\text{T}}^{\left(\text{num}\right)}$ denotes the matrix of the numerical embeddings. *W* and ${\text{B}}^{\left(\text{num}\right)}$ represent the learnable parameter matrix and bias matrix, respectively. On the other hand, for each categorical feature termed $X_{i}^{\left(cat\right)}$, we have an embedding lookup table $E_{i}(\cdot )$, for i∈{1,2,⋯,m}. The lookup table is an S×d matrix where *S* represents the number of all categories in the input table data and where *d* represents the dimension of the embedding. Each category corresponds to a unique vector. For example, assume that the representation of gender is 1 for males; then, the embedding vector for this category is the vector in the first row of the lookup table. After all the corresponding vectors in the lookup table and an added bias matrix ${\text{B}}^{\left(\text{cat}\right)}$, are obtained, the result serves as the final embedding representation for the categorical features. It can be summarized as follows:

$$T^{\left(cat\right)}=E(X^{\left(cat\right)})+B^{\left(cat\right)},$$ (3)

where ${\text{T}}^{\left(\text{cat}\right)}$ denotes the matrix of the categorical embeddings. $X^{\left(cat\right)}=[X_{1}^{\left(cat\right)},X_{2}^{\left(cat\right)},\cdots ,X_{m}^{\left(cat\right)}]$ is the matrix of raw categorical features in the input table data, and $E(X^{\left(cat\right)})=[E_{1}(X_{1}^{\left(cat\right)}),E_{2}(X_{2}^{\left(cat\right)}),\cdots ,E_{m}(X_{m}^{\left(cat\right)})]$ represents the matrix consisting of all the input corresponding vectors grabbed from the lookup table. Finally, by concatenating the embeddings of numerical and categorical features, the output of the feature embedding layer can be described as:

$$T=concat(T^{\left(num\right)},T^{\left(cat\right)})=[T_{1}^{\left(num\right)},\cdots ,T_{n}^{\left(num\right)},T_{1}^{\left(cat\right)},\cdots ,T_{m}^{\left(cat\right)}]\in {\mathbb{R}}^{d\times (n+m)}.$$ (4)

### Intrafeature interaction module

The intrafeature interaction module constitutes one of the core parts of the Strokeformer model. Combined with the interfeature interaction module, it deeply explores the interrelationships among the input feature embeddings. To effectively learn the information within a single embedding, a feedforward neural network module is designed for each feature, with its architecture shown in Fig 1. The operation process of the intrafeature interaction module is summarized as follows:

$$T_{i}^{\prime }=Linear(Dropout(ReLU(Linear(T_{i})))),$$ (5)

where *T*_*i*_ represents the input embedding of the *i*th feature and where $T_{i}^{\prime }$ denotes the output. A two-layer network structure is utilized to learn the information within each feature. *Linear* represents a linear layer operation. A *ReLU* activation function is added between the two linear layers to capture nonlinear relationships within the input embeddings. In addition, the *Dropout* mechanism is introduced to reduce overfitting and enhance the model’s generalization ability. It is placed after the *ReLU* function, before the second linear layer.

The module is specifically designed in this study to extract information within individual features, enhancing Strokeformer power on datasets containing a limited number of samples. In the implementation, because the same feedforward network structures described in Eq (5) are used for each feature, the calculations are performed in parallel to improve the model’s computational efficiency.

### Interfeature interaction module

The interfeature interaction module contains a stack of *N* encoder layers derived from the transformer. Each encoder layer consists of a multihead self-attention layer followed by a feedforward layer, with layer normalization being performed before each layer, drawing on the PreNorm model [45]. A self-attention layer comprises three parametric matrices: key, query, and value. Each input embedding is projected to these matrices to generate its key, query, and value vectors. Formally, let $K\in {\mathbb{R}}^{d\times d_{k}}$, $Q\in {\mathbb{R}}^{d\times d_{k}}$, and $V\in {\mathbb{R}}^{d\times d_{v}}$ be the matrices comprising the key, query, and value vectors of all the embeddings, respectively. *d* is the number of embeddings inputted to the self-attention layer, and *d*_*k*_ and $d_{v}$ are the dimensions of the key and value vectors, respectively. Every input embedding attends to all other embeddings through an attention head, which is computed as follows:

$$Attention(K,Q,V)=softmax((QK^{T})/\sqrt{d_{k}})\cdot V,$$ (6)

where $softmax((QK^{T})/\sqrt{d_{k}})$ (termed *A*) is called the attention matrix. For each embedding, $A\in {\mathbb{R}}^{d\times d}$ calculates how much it attends to other embeddings, thus transforming it into a contextual embedding. The output of the attention head of dimension $d_{v}$ is projected back to the embedding of dimension *d* through a fully connected structure consisting of two linear layers and the following activation function.

If $T_{i}^{\left(j\right)}$ represents the output of the *j*th transformer encoder layer, with the input $T_{i}^{\left(j-1\right)}$, j={1,2,⋯,N}. *N* represents the number of encoder layers. The computational process of the *j*th encoder layer is as follows:

$$T_{i}^{\left(j\right)}=F_{j}(T_{i}^{\left(j-1\right)})=MLP(Add\&LayerNorm(MultiHead(Add\&LayerNorm(T_{i}^{\left(j-1\right)})))),$$ (7)

where $F_{j}(\cdot )$ indicates the operation of the *j*th encoder layer, Add&LayerNorm represents a residual connection along with a layer normalization operation, *MultiHead* denotes the multihead attention operation, and *MLP* indicates the feedforward neural network module including two linear layers and an activation function between them. With *N* stacking encoder layers, the entire operating process of the interfeature interaction module is summarized as follows:

$$T_{i}^{\left(N\right)}=F_{N}(F_{N-1}\cdots F_{2}(F_{1}(T_{i}^{\prime }))).$$ (8)

The output feature matrices of the first and last layers of the transformer are denoted as ${\text{T}}^{\left(1\right)}$ and ${\text{T}}^{\left(\text{N}\right)}$, respectively. The final *Mean* operation, referring to averaging along the first dimension of the tensor, is given by:

$$T^{\left(out\right)}=Mean(T^{\left(1\right)},T^{\left(N\right)}),$$ (9)

where ${\text{T}}^{\left(\text{out}\right)}$ represents the final output of the interfeature interaction module.

This module is designed to utilize the power of the transformer structure. Layer normalization and residual connections are introduced to ensure that the transformer layers converge. The module works as a main part of the proposed Strokeformer encoder, which aims to capture the information between the features effectively.

### Output layer

For the output matrix ${\text{T}}^{\left(\text{out}\right)}$ from the feature interaction layer, the final classification result of the Strokeformer model is obtained by introducing a linear layer in the output layer, whose operation is described as follows:

$$\hat{y}=Linear(ReLU(LayerNorm(T^{\left(out\right)}))),$$ (10)

where $\hat{y}$ represents the final predictive output of the Strokeformer model. The output dimension of the *Linear* layer in Eq (10) is one, indicating the probability that the model gives its prediction result as the positive class. Typically, a sigmoid function is used to constrain the final output in the range between 0 and 1, and a threshold of 0.5 is set. When the output probability is equal to or greater than the threshold, the prediction is positive; otherwise, the prediction is negative. Binary cross-entropy (BCE) with a logit loss function is used to calculate the error between the predicted value $\hat{y}$ and the sample label *y*. This loss function simultaneously conducts the sigmoid transformation and the computation of the BCE.

## Learning paradigm

Strokeformer can be trained in an end-to-end supervised manner via labelled examples. However, to address the overfitting problem, the proposed training method comprises a self-supervised pretraining process to learn feature representations from unlabelled data, followed by a fine-tuning process on a target-supervised task.

**Fig 2:**
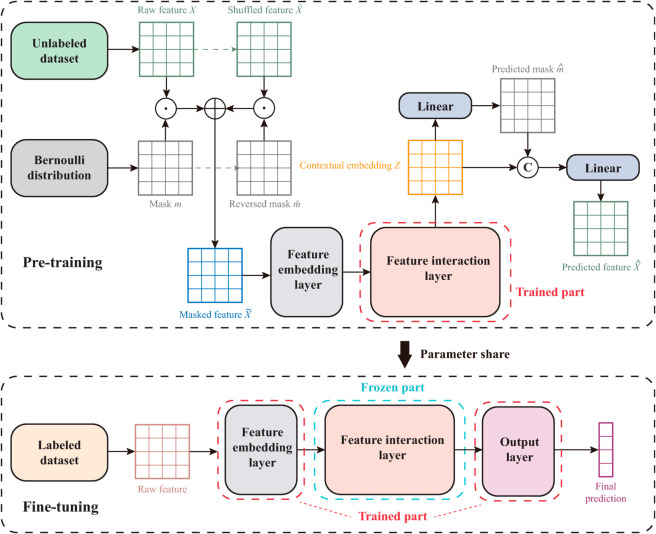
Pretraining and fine-tuning paradigm for the Strokeformer model. In the pretraining phase, the feature interaction layer is trained on a large-scale, unlabelled dataset from another corresponding area via self-supervised learning. In the fine-tuning phase, the parameters in the feature interaction layer are frozen, and the feature embedding layer and the output layer are trained on the small-scale target dataset via supervised gradient-based learning.

As shown in Fig 2, two prediction tasks, namely, predicting the masked features and determining whether the features are masked, are employed in the pretraining phase. First, mask matrices are randomly initialized, and the original input features are masked to obtain the masked features. Then, the masked features are processed through the feature embedding layer and the feature interaction layer of the Strokeformer model, and different additional linear layers are used to separately predict the mask matrix and the masked features. Finally, the mask loss and feature loss are summed to obtain the final loss value, which is minimized to train the model. The pretraining process uses a large amount of unlabelled data to conduct self-supervised learning, enabling the model to learn correlations among features. After that, the parameters of the pretrained model are reused and trained on a smaller labelled dataset in the fine-tuning phase.

Owing to the difficulties in obtaining labelled clinical data, the stroke prognosis challenge naturally faces the problems of an insufficient number of samples and class imbalance in the dataset. The learning paradigm, consisting of pretraining and fine-tuning, is designed to handle these problems. A large-scale dataset with well-balanced samples is employed in the pretraining stage, which is believed to help reduce the overfitting problem caused by the limited sample counts and imbalance existing in the target medical dataset. Details of the implementation of the pretraining and fine-tuning processes are described in the following sections.

### Pretraining

Let *m* denote the binary mask matrix and *g*_*mask*_ denote the masked feature generation function. The process of obtaining the masked features from the original features can be represented as:

$$\tilde{X}=g_{mask}(X,m)=m⊙\bar{X}+(1-m)⊙X,$$ (11)

where $X\in {\mathbb{R}}^{d\times k}$ signifies the original feature matrix, $\bar{X}\in {\mathbb{R}}^{d\times k}$ refers to the feature matrix obtained by randomly shuffling the order of samples in the original feature matrix *X*, and $\tilde{X}\in {\mathbb{R}}^{d\times k}$ represents the masked feature matrix. *k* represents the number of samples, and *d* indicates the feature dimension. Each $m_{ij}\in \{0,1\}$ (i={1,2,⋯,d},j={1,2,⋯,k}) in *m* is generated from a Bernoulli distribution, and *m*_*ij*_ = 1 indicates that the feature at the corresponding position of the sample is masked.

The masked feature matrix $\tilde{X}$ is fed to the former part of Strokeformer (feature embedding layer and feature interaction layer, termed *s*) to yield the output *Z*. The two tasks adopted by pretraining, i.e., predicting the masked features and whether the features are masked, can be depicted as follows:

$$\left\{ \begin{array}{c} \hat{m}=g_{m}(Z),\\ \hat{X}=g_{f}(concat(Z,\hat{m})), \end{array}\right.$$ (12)

where $\hat{m}$ represents the predicted mask matrix, *g*_*m*_ denotes the linear layer used for predicting the mask matrix, $\hat{X}$ represents the predicted features, and *g*_*f*_ indicates the linear layer utilized for predicting the original features.

If *s* refers to the former part of the Strokeformer model without the final classifier, the parameters *s*, *g*_*f*_, and *g*_*m*_ are adjusted through backpropagation by computing loss values. The entire process of pretraining can be represented as:

$$\min_{s,g_{f},g_{m}}{𝔼}_{X∼p_{X},m∼p_{m},\tilde{X}∼g_{mask}(X,m)}[L_{m}(m,\hat{m})+\alpha L_{f}(X,\hat{X})],$$ (13)

where ${𝔼}_{X∼p_{X},m\sim p_{m},\tilde{X}\sim g_{mask}(X,m)}$ represents the calculated expected value of the loss function, *p*_*X*_ is the probability distribution of the original features, *p*_*m*_ is the Bernoulli distribution utilized to generate the mask matrix, *α* indicates the weight coefficients, $L_{m}(m,\hat{m})$ denotes the BCE loss function for predicting the mask matrix, and $L_{f}(X,\hat{X})$ signifies the loss function for predicting features, mean squared error for numerical features and cross-entropy for categorical features.

### Fine-tuning

In this stage, the parameters from the transformer encoder layers of the pretrained model are reused and frozen during the supervised learning process. Consequently, the primary focus of training in the fine-tuning phase is on the embedding layer, which generates the embeddings of the input features, and the output layer, which provides the final predictions. The optimization process of fine-tuning is summarized as follows:

$$\min_{e,g}{𝔼}_{(X,y)\sim p_{Xy}}[L_{s}(y,g(f(e(X))))],$$ (14)

where ${𝔼}_{(X,y)\sim p_{Xy}}$ represents the expectation of the loss function. *X* denotes the input features, *y* represents the labels, and (*X*,*y*) samples according to the joint probability distribution *p*_*Xy*_. *L*_*s*_ is the BCE loss function. *e* represents the feature embedding layer, *f* indicates the feature interaction layer, and *g* is the output layer that provides the predicted label values. In addition, an early stopping strategy [46] was incorporated during the model training phase to prevent overfitting.

## Experiments

In this section, first, 22 introduced binary classification datasets and five utilized performance metrics are described in the subsections Datasets descriptions and Performance metrics, respectively. More details about the experimental design and setup are presented in the Experimental Setup subsection. Finally, in this subsection, compared with those of other stroke prognosis prediction methods, the classification performances of the proposed Strokeformer model and the training algorithm are compared against those of several state-of-the-art machine learning methods and deep learning-based methods that are specially developed for performing classification on tabular data.

### Dataset description

To investigate the classification performance of the proposed model and training algorithm, 19 benchmark datasets sourced from OpenML [47] and two private stroke prognosis datasets provided by Shenzhen Fuyong People’s Hospital (termed the Fuyong dataset) and The Affiliated Taizhou People’s Hospital of Nanjing Medical University (termed the Taizhou dataset) are used in our experiments. In addition, the pretraining phase of the proposed learning algorithm uses another open-source dataset called the Covertype dataset, which is also publicly available from OpenML. Table 1 summarizes the number of samples, the number of positive and negative samples, the number of attributes, and the attribute characteristics of all the introduced datasets.

**Table 1:** Description of the employed classification datasets.

Dataset	Instances	Positive instances	Negative instances	Features	Numberical features	Categorical features
Covertype	432680	211840	211480	54	44	10
Blood	748	178	570	4	4	0
Breast-w	683	444	239	9	9	0
Credit-A	653	296	357	15	6	9
Diabetes	768	268	500	8	8	0
Ilpd	583	167	416	10	9	1
Kc2	522	107	415	21	21	0
Qsar	1055	356	699	41	41	0
Tic	958	626	332	9	0	9
Wdbc	569	212	357	30	30	0
Albert	58251	29125	29126	31	21	10
Bank	41188	4640	36548	20	10	10
California	20634	10317	10317	8	8	0
Compas	4966	2483	2483	11	3	8
Credit	13272	6636	6636	21	20	1
Customer	7032	1869	5163	19	3	16
Electricity	38474	19237	19237	8	7	1
Eye	7608	3804	3804	23	20	3
House	13488	6744	6744	16	16	0
Income	32561	7841	24720	13	5	8
Fuyong	117	23	94	14	6	8
Taizhou	248	99	185	14	2	12

In this study, the 19 introduced benchmarks are divided into two subsets: nine small-scale datasets and ten additional datasets with relatively large scales. On the one hand, as shown in Table 1, the number of instances of the small-scale datasets is several hundred, and the number of features varies across these datasets, ranging from 4–41. Notably, Credit-A and Ilpd contain both numerical and categorical features, Tic has only categorical features, and the other datasets include only numerical features. Serious imbalances can be observed in datasets such as Blood and Kc2. On the other hand, the large-scale datasets span a broad range of sample counts, varying from 4,966 to 58,252, and incorporate a mix of categorical and numerical features. The feature counts of these datasets range from 8–31. Table 1 shows that the Bank, Customer, and Income datasets present imbalanced scenarios, with positive-to-negative ratios of 1:7, 1:2, and 1:3, respectively. In contrast, the remaining seven datasets exhibit a balanced ratio of positive to negative samples.

The labelled medical datasets, such as stroke prognosis records, are insufficient. This is because collecting and labelling these records typically requires substantial time and effort from domain experts. Therefore, the training paradigm of performing pretraining on large-scale unlabelled datasets from other domains and then using small-scale labelled medical datasets for fine-tuning is investigated in this study. The Covertype dataset introduced in our pretraining is provided by the United States Geological Survey and the United States Forest Service to predict forest cover types based on cartographic data. It comprises ten numerical attributes, such as elevation, azimuth, and slope, along with 44 categorical attributes, encompassing four wilderness areas and 40 soil types. There are seven types of forest cover, including spruce/fir, pole pine, ponderosa, and pine, among others. This study selects spruce/fir and lodgepole pine for a binary classification task. The experimental results demonstrated the effectiveness of the pretraining process, which was conducted on data from other domains.

**Fig 3:**
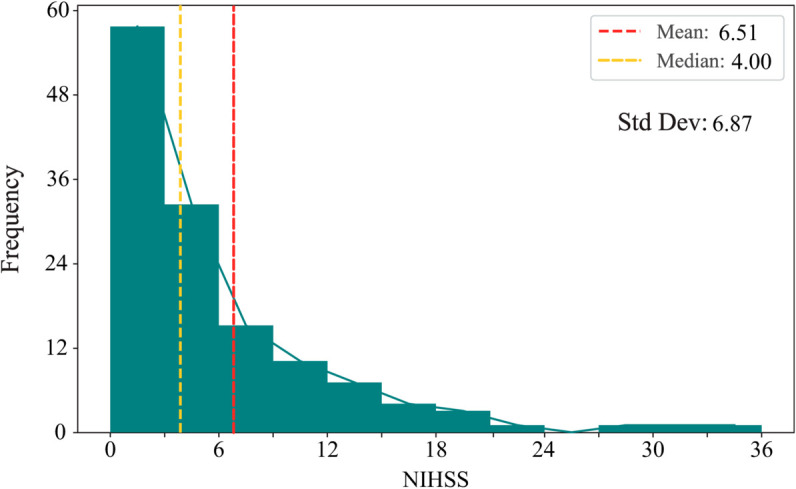
Prethrombolysis NIHSS score distribution in the Fuyong dataset.

**Fig 4:**
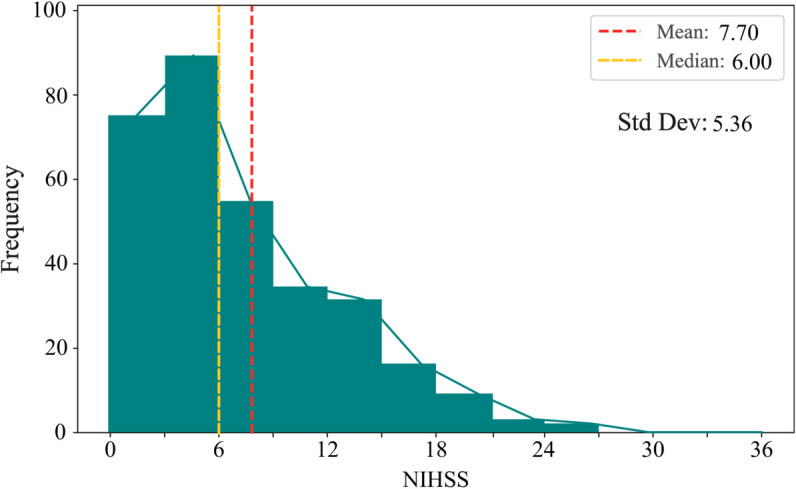
Prethrombolysis NIHSS score distribution on the Taizhou dataset.

Finally, two private stroke prognosis datasets are adopted to estimate the performance of the proposed methods on real-world tasks. The Fuyong dataset contains records of 117 stroke patients who received treatment at Shenzhen Fuyong People’s Hospital between March 16, 2022, and August 27, 2024. In addition, the medical records of 248 stroke patients treated at The Affiliated Taizhou People’s Hospital of Nanjing Medical University between January 1, 2020, and December 31, 2023, were included in the Taizhou dataset. The two private stroke prognosis datasets meticulously track symptoms and their changes in patients before and after thrombolysis therapy, encompassing variables such as patient age, sex, prethrombolysis National Institutes of Health Stroke Scale (NIHSS) score [48], postthrombolysis NIHSS score, the presence of hypertension, atrial fibrillation, and diabetes, among other clinical indicators. Notably, the NIHSS score is a crucial metric used to gauge the severity of a stroke, with lower scores indicating milder strokes. This study defines patients whose NIHSS score decreases by four points or more after thrombolysis as positive samples; otherwise, they are considered negative samples. Samples containing missing values are discarded during the preprocessing phase. The datasets exhibit class imbalance, with a ratio of approximately 1:4.1 between positive and negative samples in the Fuyong dataset and 1:1.9 in the Taizhou dataset. The distributions of NIHSS scores from 0 to 36 among patients recorded in the Fuyong and Taizhou datasets are shown in Figs 3 and 4, respectively. The apparent leftward skew observed in both datasets indicates that milder stroke cases are much more common than severe cases. The red and yellow dashed lines mark the mean and median scores, respectively, in the figures, implying the severely skewed nature of the two datasets. Furthermore, the standard deviations are also shown in the figures. The heterogeneity of the two real-world stroke datasets is emphasized in these visualizations.

### Performance metrics

To evaluate the classification performance of the proposed Strokeformer model comprehensively, five widely used classification metrics for binary classification tasks are employed, including accuracy (*Acc*), precision (*Pre*), recall (*RCL*), F-measure (*F*1), and area under the receiver operating characteristic curve (*AUC*) [49]. Their mathematical expressions are given as follows:

$$Acc=\frac{TP+TN}{TP+TN+FP+FN},$$ (15)

$$Pre=\frac{TP}{TP+FP},$$ (16)

$$RCL=\frac{TP}{TP+FN},$$ (17)

$$F1=\frac{2TP}{2TP+FP+FN},$$ (18)

$$AUC=\int_{0}^{1}\frac{TP}{TP+FN}d\frac{FP}{TN+FP},$$ (19)

where *TP* and *FN* indicate the number of correctly classified positive and negative samples, respectively, and where *FP* and *FN* represent the number of instances misclassified as positive and negative, respectively. *Acc* measures the proportion of correctly predicted samples out of all the records, offering a general assessment of classification performance. However, in cases of imbalanced data distribution, *Acc* alone fails to fully reflect the actual performance of the model. *Pre*, also referred to as the positive predictive value, represents the ratio of correctly predicted positive samples to all samples predicted as positive. In the context of stroke prognosis prediction, *Pre* is particularly crucial. Compared with failure to administer thrombolytic therapy in a timely manner to eligible patients, mistakenly applying thrombolytic treatment to those who do not meet the criteria poses a greater risk. Incorrect treatment decisions may subject patients to unnecessary health risks, especially for those with contraindications, where inappropriate thrombolytic therapy could lead to serious, potentially fatal bleeding events, worsening the patient’s prognosis. *RCL*, also known as sensitivity, indicates the proportion of true positives correctly identified and reflects the model’s ability to correctly identify positive instances from the actual positives. *Pre* and *RCL* often exhibit a trade-off relationship. The *F*1 score is designed to provide a comprehensive performance indicator through the harmonic mean of *Pre* and *RCL*. By balancing *Pre* and *RCL*, it has become a significant metric for assessing performance in complex prediction tasks. In this study, *AUC* was selected as the core metric to measure performance. This is because *AUC* quantifies the overall ability of the model to accurately discriminate between positive and negative samples across various decision thresholds, with values closer to 1 indicating better model performance. Therefore, *AUC* is not sensitive to class imbalance.

### Experimental setup

Several state-of-the-art methods, including the machine learning techniques XGBoost and CatBoost and deep learning methods such as ARMNet, TabNet, TabTransformer, and FT-Transformer, are introduced as competitors of the proposed Strokeformer model. The proposed Strokeformer model is separately trained by direct stochastic gradient descent (SGD) on the raw training set (termed the Strokeformer_*SGD*_), SGD with the additional data augmentation technique Mixup [50] (Strokeformer_*SGD* + *M*_), and the described framework contains pretraining (Strokeformer_*PT*_) and fine-tuning (Strokeformer_*FT*_). Mixup is a data augmentation technique aimed at enhancing the generalization capability of deep learning models across various tasks [51,52]. To ensure optimal model performance across different datasets, the hyperparameters were adjusted accordingly. Table 2 details the adjustable ranges of hyperparameters used for each model in the experiments.

**Table 2:** Hyperparameter settings of the machine learning and deep learning methods.

Models	Hyperparameter
XGBoost	n_estimators=100, lr=0.1, max_depth=4, eval_metric=“Logloss”
CatBoost	n_estimators=100, lr=0.1, max_depth=4, eval_metric=“Logloss”
ARMNet	n_emb=128, alpha=[1.5, 1.7, 2.0], n_hid=128, d_k=128, mlp_nlayer=1, mlp_hid=3
TabNet	default
TabTransformer	dim=32, depth=6, heads=8, lr=$2\times 10^{-4}$, optimer=Adam
FT-Transformer	d_token=[96, 128, 192, 256, 320, 384], head_num=8, n_blocks=[1, 2, 3, 4, 5, 6], lr=$2\times 10^{-4}$, optimer=Adam
Strokeformer_*SGD*_	d_token=[96, 128, 192, 256, 320], head_num=8, n_blocks=[1, 2, 3, 4, 5], lr=$2\;\times \;10^{-4}$, optimer=Adam
Strokeformer_*PT*_	p_m=0.5, batch_size=256, epoch=30, lr=$2\times 10^{-4}$, *α*=2, *β*=3, *γ*=0.75, d_token=192, head_num=8, n_blocks=3, optimer=Adam
Strokeformer_*FT*_	batch_size=256, lr=$2\;\times \;10^{-4}$, d_token=192, head_num=8, n_blocks=3, optimer=Adam

In addition, to ensure the stability and reliability of the results, a ten-fold cross-validation [53] approach is employed for model training on each dataset, with training, validation, and test sets comprising 72%, 18%, and 10% of the data, respectively. The ten-fold cross-validation was repeated three times for each dataset to further validate model performance, resulting in 30 sets of model predictions. The final prediction for each dataset is obtained by averaging these predictions.

### Comparison to other stroke prognosis prediction methods

**Fig 5:**
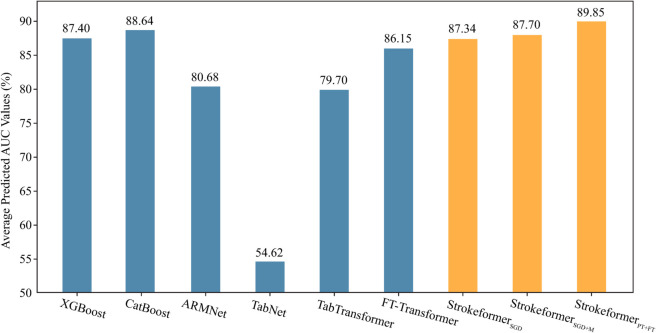
Performance comparison in terms of the average predicted AUC of the introduced methods on nine small-scale datasets.

**Experiments on nine small-scale benchmark datasets**. The performances of Strokeformer trained by SGD (Strokeformer_*SGD*_), by SGD with Mixup (Strokeformer_*SGD* + *M*_), and by the pretraining and fine-tuning strategies (Strokeformer_*PT* + *FT*_) on the nine small-scale binary datasets are evaluated in this section. The results in terms of the AUC of the three methods and all other introduced competitors are presented in Table 3. The table shoes that Strokeformer_*PT* + *FT*_ outperforms all the other competitors on seven datasets: Blood, Breast, Credit-A, Diabetes, Kc2, Qsar, and Wdbc. For the remaining two datasets Ilpd and Tic, on Ilpd, the proposed Strokeformer_*SGD* + *M*_ ranks first, and the performance of Strokeformer_*PT* + *FT*_ is slightly worse than that of the other methods (less than 0.5%). All the employed models except for TabNet achieve quite high AUCs on Tic, and the differences among them are insignificant. The proposed Strokeformer_*SGD*+*M*_ and Strokeformer_*PT*+*FT*_ both derived AUCs over 99%. Furthermore, this study focuses on the average predicted AUC across all test datasets because the diversity of dataset distributions makes it challenging for any single model to achieve optimal results in all datasets. Fig 5 shows the average prediction AUC for each model across nine small-scale datasets, revealing that the Strokeformer_*PT*+*FT*_ method significantly outperforms the other methods in terms of average predictive performance. Notably, the average prediction AUC of the Strokeformer_*PT*+*FT*_ model clearly outperforms that of the FT-Transformer model, indicating that introducing the internal feature interaction module after the embedding layer in the proposed Strokeformer model to learn information within individual features can effectively enhance the predictive performance of the model. A comparison between Strokeformer_*SGD*_ and Strokeformer_*PT*+*FT*_ proves that the pretraining and fine-tuning approaches significantly improve the model’s generalizability and enhance its performance on small-scale datasets. The performance of the Strokeformer_*SGD*+*M*_ method does not surpass that of the Strokeformer_*PT*+*FT*_, possibly because the Mixup method is applicable only to numerical features and is not proper for categorical features. In contrast, the pretraining plus fine-tuning approach in the Strokeformer_*PT*+*FT*_ can more effectively account for the characteristics of both numerical and categorical features.

**Table 3:** Prediction AUC of Strokeformer and the competitor methods on the nine small-scale binary classification datasets.

Models	Datasets								
	Blood	Breast	Credit-A	Diabetes	Ilpd	Kc2	Qsar	Tic	Wdbc
XGBoost [16]	70.85	99.30	93.58	77.31	70.61	83.24	92.63	99.93	99.17
CatBoost [38]	73.44	99.31	94.02	84.03	72.71	82.75	92.50	**99.98**	99.04
ARMNet [39]	71.76	93.74	79.76	75.13	64.48	70.78	88.75	90.68	91.00
TabNet [39]	52.78	79.07	53.65	51.31	47.18	48.32	45.34	45.17	68.78
TabTransformer [32]	49.85	94.91	92.18	62.30	51.77	80.67	91.28	99.87	94.47
FT-Transformer [44]	63.57	98.93	91.19	79.31	71.96	81.92	91.83	99.36	97.29
Strokeformer_*SGD*_	71.08	99.10	93.42	80.28	72.32	82.40	91.95	98.62	96.93
Strokeformer_*SGD* + *M*_	71.57	99.36	93.50	80.99	**72.74**	82.10	91.91	99.28	97.88
Strokeformer_*PT* + *FT*_	**78.24**	**99.66**	**94.20**	**87.84**	72.42	**83.67**	**93.39**	99.22	**99.98**

**Fig 6:**
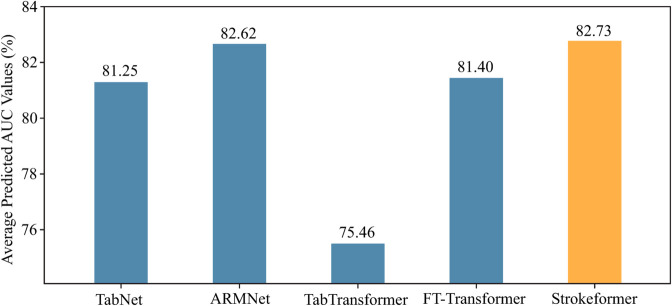
Performance comparison in terms of the average predicted AUC of the introduced methods on ten large-scale datasets.

**Experiments on ten large-scale benchmark datasets**. To further explore the predictive performance of Strokeformer for binary classification problems, this experiment not only examined datasets with fewer than 2,000 test samples but also specifically selected ten relatively large-scale datasets with more than 2,000 samples for testing. The experimental results are shown in Table 4. In this experiment, Strokeformer is trained directly by SGD because the Mixup method and pretraining plus fine-tuning training paradigm are designed to address the problem of insufficient training data when the large-scale dataset contains sufficient labelled samples. The proposed model outperforms all the competitors on seven datasets: Albert, Bank, Compas, Customer, Electricity, Eye, and Income. ARMNet appears to perform better on large-scale datasets; it achieves a better prediction AUC than do all other models on California, Credit, and House. However, Strokeformer still performs better on all the testing datasets than TabTransformer and FT-Transformer do, which indicates that the proposed model achieves state-of-the-art performance among all the transformer-based models in dealing with tabular data. In addition, as shown in Table 1, the California, Credit, and House datasets, on which ARMNet outperforms Strokeformer, are all class balanced and contain equal numbers of positive and negative samples. In contrast, the Bank, Customer, and Income datasets include three class-imbalanced cases. According to the results presented in Table 4, Strokeformer achieves better classification performance on these datasets than ARMNet and other competitors do. These experimental results demonstrate the advantages of Strokeformer on class-imbalanced datasets. The average prediction AUC shown in Fig 6 also supports the superiority of Strokeformer in large-scale datasets; it outperforms all other comparative models. Relative to small sample datasets, the ARMNet model performed better on large-scale datasets, with its predictive performance approaching that of the Strokeformer model.

**Table 4:** Prediction AUC of Strokeformer and the competitor methods on the ten large-scale binary classification datasets.

Models	Datasets									
	Albert	Bank	California	Compas	Credit	Customer	Electricity	Eye	House	Income
ARMNet [39]	71.36	94.18	**93.42**	72.88	**77.74**	84.37	86.38	60.72	**94.06**	91.07
TabNet [39]	69.48	93.95	93.01	69.05	76.78	81.99	88.21	59.84	89.69	90.49
TabTransformer [32]	68.94	91.77	74.22	69.59	66.27	82.90	85.52	55.36	72.04	87.96
FT-Transformer [44]	71.46	94.09	89.26	73.14	75.56	84.40	90.35	52.10	92.10	91.57
Strokeformer	**71.70**	**94.33**	89.82	**73.18**	76.70	**84.58**	**91.05**	**61.25**	93.05	**91.60**

**Fig 7:**
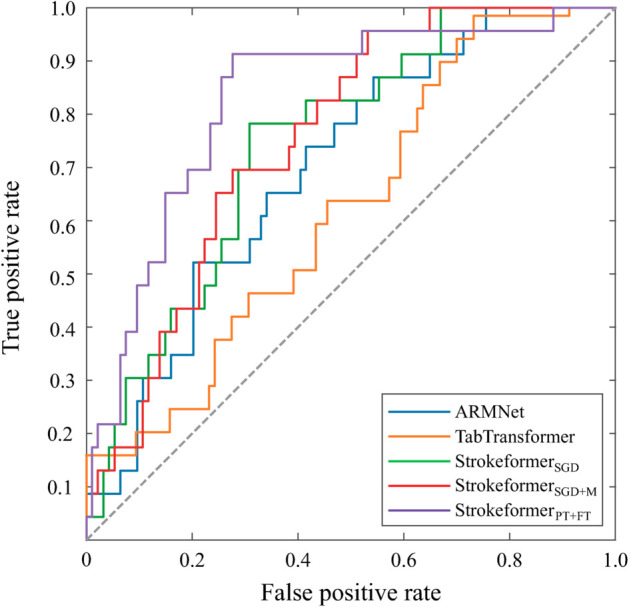
ROC curves of the proposed Strokeformer model and its competitors on the private Fuyong stroke prognosis dataset.

**Fig 8:**
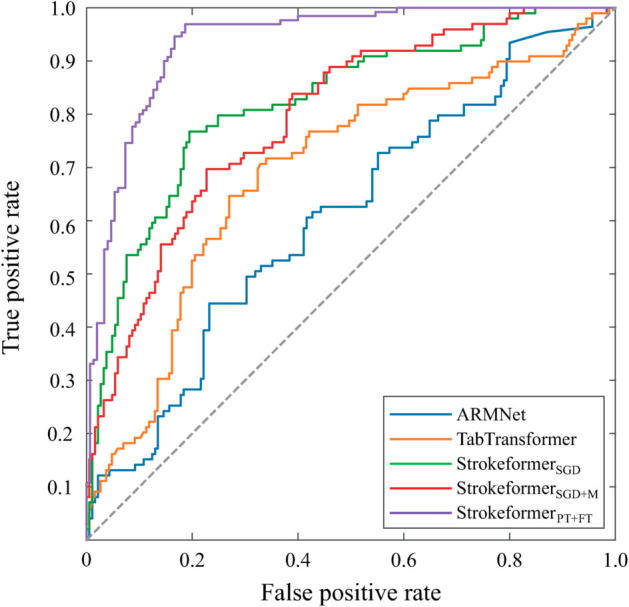
ROC curves of the proposed Strokeformer model and its competitors on the private Taizhou stroke prognosis dataset.

**Experiments on two real-world stroke prognosis datasets.** Through these experiments, the excellent predictive performance of the Strokeformer model on tabular data classification problems has been adequately verified. This section applies the model to real-world stroke prognosis prediction tasks using the private Fuyong and Taizhou datasets. The experimental results evaluated across multiple performance metrics, such as accuracy, recall, precision, and F1 score, are shown in Table 5 and Table 6. The results indicate that Strokeformer_*PT* + *FT*_ achieves an AUC that significantly outperforms the predictive capabilities of other comparative models. Strokeformer also outperforms other competitor models in terms of other predictive metrics, such as accuracy and precision. The predicted AUC of Strokeformer_*PT* + *FT*_ reached 92.79%, an improvement of 8.57% compared with the 84.22% prediction result from Strokeformer_*SGD*_ on the Taizhou dataset, highlighting the significant performance enhancement derived by using pretraining and fine-tuning methods. Fig 7 and Fig 8 display the ROC curves for deep learning models such as ARMNet and TabTransformer and the proposed Strokeformer model on the Fuyong dataset and Taizhou dataset, respectively. The figure clearly shows the significant superiority of Strokeformer_*PT* + *FT*_. As presented in Eqs (17) and (18), the F1 and Recall values are highly dependent on the number of correctly classified positive samples; they appear low on the Fuyong dataset because of its small size and class imbalance, containing only 23 positive instances and 94 negative instances. As shown in Table 5, TabNet achieves the highest Recall value; however, its accuracy and AUC are very low, indicating that the model fails to converge on this dataset. In such a case, a high Recall value is considered incidental and unreliable. Similarly, although ARMNet has relatively high Recall and F1 values, its low accuracy and AUC suggest that it does not perform well on the Fuyong dataset. The performance improvement of Strokeformer on the stroke prognosis dataset is more pronounced than that on the other datasets because the pretraining and fine-tuning processes greatly alleviate the overfitting and performance degeneration caused by class imbalance in the training set. Our assumption that using data from other domains for pretraining can improve the classification performance of some small-scale medical datasets is correct.

**Table 5:** Comparisons among the proposed Strokeformer model and the state-of-the-art competitors on the private Fuyong stroke prognosis dataset.

Methods	Accuracy	Recall	Precision	F1	AUC
ARMNet [39]	70.94	33.33	28.68	**29.90**	68.59
TabNet [40]	24.79	**78.26**	17.82	29.03	49.26
TabTransformer [32]	80.06	0.00	0.00	0.00	57.52
FT-Transformer [44]	78.63	10.15	33.97	15.09	66.79
Strokeformer_*SGD*_	77.78	11.59	31.09	16.33	67.88
Strokeformer_*SGD* + *M*_	73.34	3.33	25.00	5.88	63.40
Strokeformer_*PT* + *FT*_	**80.03**	15.56	**38.33**	18.69	**71.62**

**Table 6:** Comparisons among the proposed Strokeformer model and the state-of-the-art competitors on the private Taizhou stroke prognosis dataset.

Methods	Accuracy	Recall	Precision	F1	AUC
XGBoost [16]	77.46	64.65	68.82	66.67	82.69
CatBoost [38]	79.81	67.01	72.90	69.80	85.29
ARMNet [39]	61.62	41.41	46.61	43.23	61.97
TabNet [40]	35.21	**96.97**	34.66	51.06	55.74
TabTransformer [32]	65.73	30.30	51.41	38.11	69.09
FT-Transformer [44]	78.40	68.69	69.20	68.92	83.04
Strokeformer_*SGD*_	80.75	73.07	72.19	72.59	84.22
Strokeformer_*SGD* + *M*_	80.52	70.37	72.82	71.57	84.61
Strokeformer_*PT* + *FT*_	**85.95**	84.87	**84.84**	**84.81**	**92.79**

## Discussion

In this section, an ablation study is discussed, which removes the intra- and interfeature interaction modules from Strokeformer separately. The experiments compare the performance of the model before and after the removal of the two modules on ten small-scale datasets to analyse the impact of each module on the ultimate prediction outcomes. Additionally, the influence of varying encoder layer counts and different feature representation methods on predictive performance is assessed. Three distinct representation strategies, adding a classify token (CLS token) [54] to the input features and using its associated feature, employing the average of the last encoder layer’s feature embeddings, and utilizing the mean of the first and last encoder layer embeddings, are investigated.

### Ablation study

To evaluate the effectiveness of each component in the Strokeformer model, ablation experiments were conducted on the intra- and interfeature interaction modules. The experiments aimed to elucidate the impact of these critical components on the final predictive performance of the model. By individually removing the intrafeature interaction module and the interfeature interaction module and observing changes in the prediction outcomes before and after removal, the contribution of each module to Strokeformer can be assessed. The experimental results on nine small-scale datasets are presented in Table 7, where ‘w/o intrafeature’ denotes removing the intrafeature interaction module and ‘w/o interfeature’ indicates the removal of the interfeature interaction module. On the one hand, the results suggest that after removing the intrafeature interaction module, a decline in performance is observed in seven out of the ten datasets, which aligns with expectations and further confirms the effectiveness of the intrafeature interaction module and its significant contribution to the Strokeformer model. However, performance improvements are noted in the Tic and Wdbc datasets. The assumption is that in these two datasets, adding the intrafeature interaction module not only fails to capture effective internal feature information but also increases model complexity, leading to overfitting. The overfitting problem can be addressed by introducing a pretraining and fine-tuning learning strategy. On the other hand, the performance decreases across all nine datasets when the interfeature interaction is removed, underscoring the critical role of the transformer encoder module in the feature processing process. Overall, the removal of either the intrafeature interaction module or the interfeature interaction module from the model leads to a certain degree of decline in the predictive performance. The results of the ablation experiment prove the effectiveness of both the intrafeature interaction module and the interfeature interaction module in the design of the proposed Strokeformer model.

**Table 7:** Prediction AUC from ablation experiments of Strokeformer on nine small-scale datasets.

Datasets	Strokeformer w/o intra-feautre	Strokeformer w/o inter-feature	Strokeformer
Blood	69.33	63.57	**71.08**
Breast	98.88	98.92	**99.10**
Credit-A	87.89	91.19	**93.42**
Diabetes	79.37	79.17	**80.28**
Ilpd	70.71	71.85	**72.32**
Kc2	81.88	79.66	**82.40**
Qsar	91.56	91.52	**91.95**
Tic	**99.62**	99.31	98.62
Wdbc	**98.17**	97.29	96.96

### Variants sensitivity analysis

The experimental results of Strokeformer with different variants on the eight small-scale datasets are presented in Table 8. Among the three compared representation methods, ‘first-last-avg’ exhibits superior performance in four out of eight datasets: Blood, Credit-A, Diabetes, and Qsar. Moreover, the ‘last-avg’ method notably stands out in the Breast, Ilpd, and Wdbc datasets. The remaining Kc2 and Tic datasets show exceptional performance in the variant that includes the CLS token. According to these results, the ‘first–last–avg’ method generally displays the best performance among all three compared variants, although the differences between these variants are not substantial. Furthermore, the experiment explores the performance changes of three different representation methods as the number of transformer layers increases from one to five. To demonstrate the trend of model prediction performance with increasing number of encoder layers. That there are variations in performance when the model changes across different datasets is clear. The trends can be roughly categorized into three types. First, there is an overall upwards trend in model performance as the number of layers increases, which is evident on datasets such as Breast and Wdbc. The second trend shows an initial rise in model performance with increasing layers, reaching an optimal point before declining as more layers are added. This trend is observed in the Credit-A, Kc2, Ilpd, and Diabetes datasets. Finally, the last trend is characterized by an overall decrease in performance as the number of layers increases, with the best performance achieved at just one layer. This can be observed in the Blood and Qsar datasets. Typically, when the number of encoder layers in a model is increased, if a dataset achieves its optimal performance with few layers, the relationships between the features are relatively simple. When the layer count of the model becomes too high, it may lead to an overly complex network, which counteracts the performance enhancement.

**Table 8:** Prediction AUC of different variants on eight small-scale datasets.

Datasets	Encoder Layers	Variants of Strokeformer		
		Last_avg	+CLS	first_last_avg
Blood	layer=1 layer=2 layer=3 layer=4 layer=5	65.67 64.40 68.98 69.29 69.19	64.13 65.23 64.73 65.31 67.43	61.62 67.17 69.42 70.87 **71.08**
Breast	layer=1 layer=2 layer=3 layer=4 layer=5	98.97 98.93 99.20 99.03 99.02	99.00 98.92 **99.91** 98.90 98.94	98.90 98.93 99.10 98.92 98.94
Credit-A	layer=1 layer=2 layer=3 layer=4 layer=5	87.53 92.50 93.25 92.56 93.31	82.09 91.13 93.08 93.30 93.25	89.66 89.30 92.46 93.14 **93.42**
Diabetes	layer=1 layer=2 layer=3 layer=4 layer=5	77.19 79.64 79.11 79.39 80.20	77.41 77.91 78.16 79.60 79.25	76.97 **80.28** 80.10 79.05 79.52
Ilpd	layer=1 layer=2 layer=3 layer=4 layer=5	69.75 **72.58** 72.07 71.88 71.81	71.41 71.84 71.12 70.87 70.80	69.37 71.66 72.32 72.29 71.60
Kc2	layer=1 layer=2 layer=3 layer=4 layer=5	81.93 81.60 81.34 81.72 80.69	**82.54** 81.37 81.69 80.95 82.29	82.40 81.32 80.72 81.17 81.64
Qsar	layer=1 layer=2 layer=3 layer=4 layer=5	91.69 91.40 91.54 91.04 91.32	91.21 91.18 91.23 91.11 91.76	**91.95** 91.59 91.41 91.23 91.25
Wdbc	layer=1 layer=2 layer=3 layer=4 layer=5	97.00 96.88 96.95 **97.07** 97.03	95.76 96.03 96.54 96.73 96.29	96.96 96.53 96.93 96.87 96.82

## Conclusions

Deep learning models have been widely applied to medical data analysis tasks, including stroke prognosis prediction. However, conventional models suffer from overfitting problems on small-scale, class-imbalanced datasets. Thus, a novel Strokeformer model is developed in this study, which overcomes these problems and achieves better performance on real-world stroke prognosis prediction tasks. The model innovatively incorporates customized intra- and interfeature interaction modules to learn information embedded within and between individual features. Furthermore, the training process of Strokeformer is extended by a pretraining and fine-tuning methodology aimed at bolstering the generalization capacity of the model. Our experimental findings reveal that the predictive performance of the proposed paradigm markedly exceeds that of other comparative methods. The primary limitation of the model lies in its lack of interpretability from the perspective of clinicians. Nevertheless, when Strokeformer is applied to the real-world Fuyong and Taizhou datasets, the model effectively increases the accuracy of stroke prognosis prediction, whereas the learning process consisting of pretraining and fine-tuning further enhances the predictive ability of the model. In summary, the proposed Strokeformer model and its particular training algorithm carry significant implications for stroke prognosis prediction, offering dependable experimental support to physicians in their diagnostic assessments of stroke prognosis. Our planned future work for this research includes two main components. First, we intend to introduce additional types of input data, such as medical images, into our model to enhance its capabilities in medical applications by integrating tabular data with other data modalities. Second, large language models are also planned to be leveraged to further improve the model’s classification performance on tabular data while expanding our focus to address more critical health care challenges beyond stroke in future work.
